# Low mindfulness is related to poor sleep quality from middle adolescents to emerging adults: a process model involving resilience and emotional dysfunction

**DOI:** 10.1186/s12888-023-05092-1

**Published:** 2023-08-28

**Authors:** Huaiyuan Zhou, Ziqing Zhu, Xiangang Feng, Ruibin Zhang

**Affiliations:** 1 Cognitive Control and Brain Healthy Laboratory, Department of Psychology, School of Public Health, Southern Medical University, Guangzhou, China; 2 Department of Psychiatry, Zhujiang Hospital, Southern Medical University, Guangzhou, China

**Keywords:** Perceived stress, Resilience, Sleep quality, Emotional dysfunction, Trait mindfulness, Middle adolescence, Emerging adult

## Abstract

**Objectives:**

Transitions from middle adolescence into merging adulthood, a life stage between age 15–25, has a high prevalence of sleep problems. Mindfulness is a trait defined as being attentive to the present moment which positively relates to sleep quality. In this study, we aimed to investigate how resilience and emotional dysfunction may influence the relationship between trait mindfulness and sleep quality.

**Methods:**

The Five Facet Mindfulness Questionnaire, Connor-Davidson Resilience Scale, Pittsburgh Sleep Quality Index and Depression Anxiety Stress Scales were used to measure the key variables through an online survey of 497 participants between middle adolescence and emerging adults (317 females, mean age 18.27 ± 0.76 years). A process model was built to investigate the mediating roles of resilience and emotional dysfunction in the impact of trait mindfulness on sleep quality, together with the relationships between their specific components.

**Results:**

We found a positive association between mindfulness and sleep quality through resilience and through emotional dysfunction, and through the sequential pathway from resilience to emotional dysfunction. Of note, acting with awareness (mindfulness facet) showed significant indirect effects on sleep quality, mediated by resilience and emotional dysfunction.

**Conclusions:**

Our findings may unveil the underlying mechanisms of how low mindfulness induces poor sleep quality. The findings indicate that conceiving mindfulness as a multifaceted construct facilitates comprehension of its components, relationships with other variables, and underscores its potential clinical significance given its critical implications for mental health.

**Supplementary Information:**

The online version contains supplementary material available at 10.1186/s12888-023-05092-1.

## Introduction

Between middle adolescence to emerging adulthood is a pre-adult period between age 15 to 25 and during which individuals are considered immature when using cognitive, emotional, and behavioural measures comparing to adults [1–3]. Therefore, when sleep is suboptimal in timing or magnitude, middle adolescents and emerging adults are more vulnerable to psychiatric disorders comparing to adults [4]. Additionally, there is a high comorbidity between psychiatric disorders and sleep problems during the vulnerable phase of childhood and adolescence [5]. Using the Pittsburgh Sleep Quality Index (PSQI), a prior study has revealed a high prevalence among emerging adults of insufficient sleep and poor sleep quality [6]. Emerging studies have indicated an underlying biological change in the circadian timing system and the homeostatic sleep regulatory process that might contribute to a sleep delay across pubertal development [3]. Also, individuals in this stage would face multiple external changes and developmental tasks in various domains (e.g., decreased parental supervision, competition in academy, intimate relationships), which may cause increased consumption of caffeine and night-use of electronics that could also delay bedtimes and risetimes. Maintaining sufficient sleep is paramount to the stable and healthy development of cognitive systems across later adolescence, especially to learning and memory [7].

Mindfulness has been described as being attentive to and aware of what is taking place at the present moment [8]. Individuals with higher mindfulness tend to focus on the present task or situation with little distraction. To date, more scholars have realized the association between trait mindfulness and sleep quality [9, 10]. Those with low mindfulness may suffer from many unwanted thoughts, thus causing a high-arousal state which significantly associates with insomnia, according to a metacognitive model of insomnia [11]. Lundh et al. (2005) also revealed that mindfulness may facilitate ‘cognitive deactivation’ which is similar to reducing arousal to mitigate sleep problems. A systematic review also provided substantial empirical evidence that mindfulness-based training may be beneficial to improving sleep quality [11].

Additionally, the sleep of adolescents and emerging adults has also been proposed to exert a negative modulatory influence on emotional regulation, which may intrigue the onset of psychiatric conditions especially anxiety and major depression [3]. Among undergraduate students, anxiety and depressive symptoms were most consistently associated with poorer sleep quality, as well as increased sleep latency, decreased sleep duration and sleep efficiency [12]. For local Chinese emerging adults, cultural differences exist such as early school time and heavy academic stress that may interact with the pubertal status to increase the risk of developing insomnia symptoms [13, 14]. Doorley et al. (2021) posited that mindfulness may be particularly effective in improving sleep quality by promoting acceptance in response to negative emotions (e.g., anxiety, stress, frustration) among individuals with chronic pain [15], which may also be particularly effective in middle adolescents and emerging adults.

Resilience was considered a strong underlying variable that affects the relationship between mindfulness and sleep quality in this study. Resilience has been described as a dynamic process encompassing positive adaptation within the context of significant adversity [16], which may play a preventive role in individuals with sleep disturbance among mid-adolescence and young adulthood [17, 18]. Resilience could mitigate the impairment of cognitive functions such as worries and ruminations [19], which might be an inducement to sleep disorders, according to Morin’s cognitive-behavioural model of insomnia [20]. Ong et al. (2012) suggested a specific metacognitive model of insomnia which proposed that the mindful attention state may allow individuals for more flexible responses toward sleep disturbance by disengaging from their daily concerns and strivings, which is constitutive of resilience. In conclusion, resilience may help adapt to the present situation, detach from unwanted thoughts and switch to normal sleep when individuals suffer from worries and ruminations in bed.

Emotional dysfunction was considered an underlying variable that affects the relationship between mindfulness and sleep quality. Emotional dysfunction is a negative psychological reaction to threats of personal life goals [21], which may have a profound negative effect particularly on the mental health of individuals between middle adolescence and emerging adulthood. According to an emotion regulation model, by changing attention deployment (resilience rather than rumination) and conducting cognitive re-appraisal (mindfulness and acceptance), emotional dysregulation would be alleviated when individuals are confronted with adversities [22, 23]. Prior studies have shown that maladaptive coping is a main predictor of emotional dysfunction and that resilience is a positive adaptation strategy which may help decrease the occurrence of negative emotions [24–26]. Yu et el. (2023) suggested a sequential mediating effect of emotional regulation and resilience on the relationship between the mindfulness component of self-compassion and college students’ depression [27]. In recent studies of somnipathy, low resilience and mood disturbance were remarkably positively correlated with sleep problems [28, 29]. Furthermore, emotional dysfunction (e.g., mood disorders including depression, anxiety, and stress) may play a vital mediating role in the association between low resilience and pre-sleep cognitive hyperarousal in subjects with insomnia [30].

Previous studies have led us to further investigations of the predetermined components of mindfulness, emotional dysfunction, and sleep quality. Sleep quality includes seven components: subjective sleep quality, sleep latency, sleep duration, habitual sleep efficiency, sleep disturbances, use of sleeping medication, and daytime dysfunction [31]. Mindfulness was studied as a multidimensional construct, including observing, describing, acting with awareness, non-judging and non-reacting [32, 33]. Baer et al. (2006) also suggest that the examination of facets of mindfulness may unveil the nature of mindfulness and its relationships with other constructs [32]. Some research studied the correlations between specific facets of mindfulness and emotional dysfunction and suggested the most significant facets of non-judging and acting with awareness [34, 35]. Fong et al. (2020) found that acting with awareness and non-reacting could predict better sleep quality in the future. Apart from that, Sala et al. (2020) implied that the five facets of mindfulness have shown a strong association with health-promoting behaviours such as physical activity, healthy eating and sleep. It has been indicated that different subtypes of emotional dysfunction (i.e., depression, anxiety, stress) can be predicted by specific facets of mindfulness [36].

**Fig. 1:**
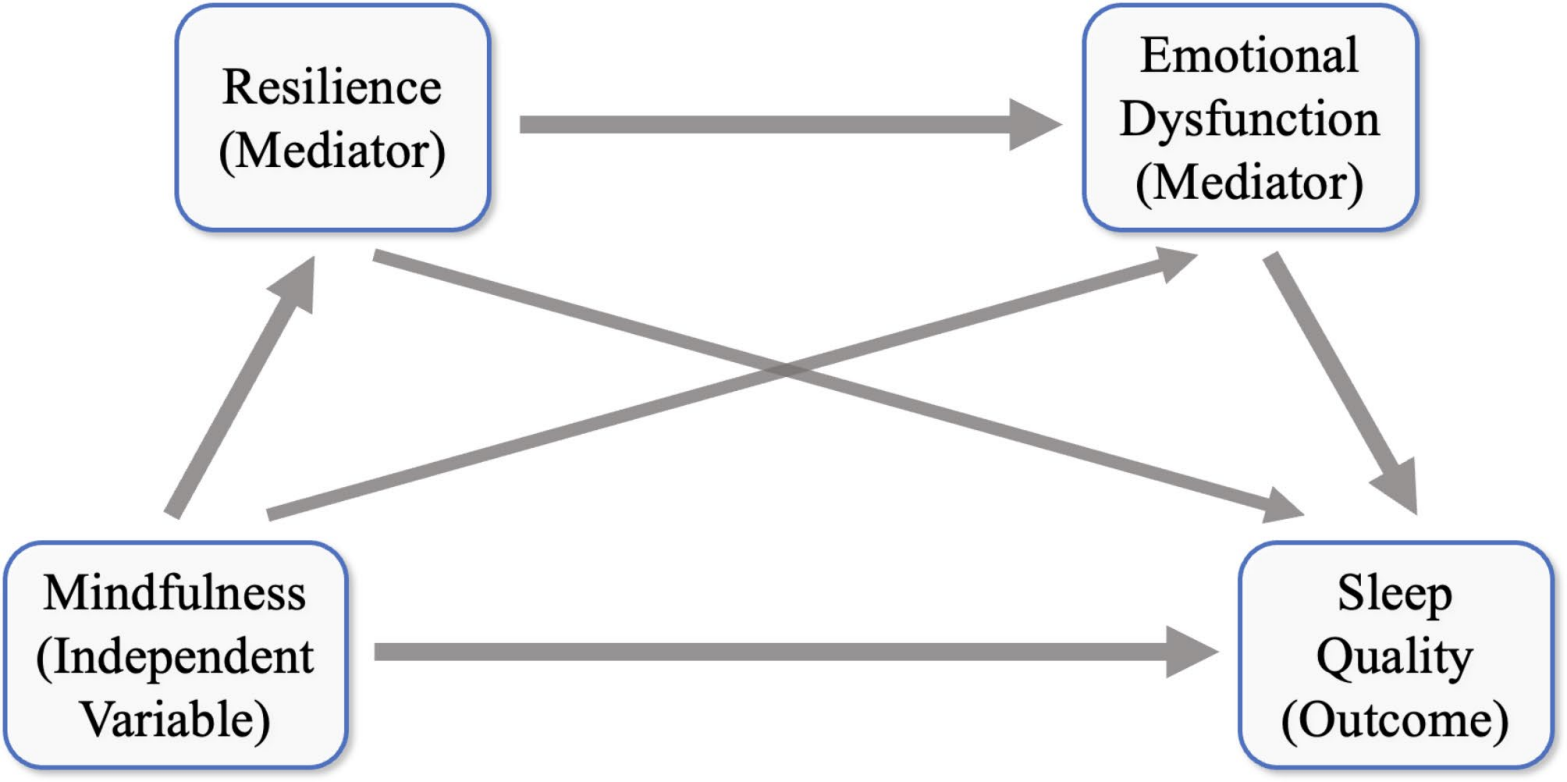
The proposed process models. Resilience and emotional dysfunction mediate the relationship between mindfulness and sleep quality separately or in sequence

The purpose of the present study was to determine whether mindfulness was associated with sleep quality and to explore the mediating effects of two variables (resilience and emotional dysfunction) in this relationship among individuals from middle adolescents to emerging adults, through a conceptual model (Fig. 1). We hypothesized that mindfulness is positively associated with sleep quality, the relationship which may also establish between specific components of the two variables. Moreover, it is also speculated that trait mindfulness may exert its influence over sleep quality through emotional states and utility of resilience, based on a sequential mediation model.

## Method

### Participants

Data were randomly collected via public advertisement from a research project investigating the risk factors of poor sleep quality among Chinese individuals from middle adolescents to emerging adults. All students were recruited through a survey platform where they voluntarily signed up for the study. A total of 634 college students enrolled in the project. The inclusion criteria were (a) currently attending the first grade, (b) aging between 15 and 25, (c) agreeing to participate in the study, (d) signing an informed consent. We excluded students from 2nd, 3rd and 4th grades since they are highly occupied by job-seeking and preparation for the post-graduate entrance examination. Additionally, we excluded participants subjects with psychiatric disorders, a history of organic brain disorder, neurological disorders, mental retardation, cerebrovascular disease, alcohol or substance abuse, pregnancy, or any physical illness. To ensure data validity, 137 cases (21.6%) were excluded during data cleaning procedures: 75 responses were considered incomplete (missing values in all items of at least one key variable, n = 31, missing values in demographic variables, n = 44) and 62 participants provided invalid (e.g., 3 to 10 h) answers about recent bedtime.

### Procedure

All materials and experimental procedures were approved by the Research Ethics Committee of the target university in Guangzhou, China. Participants completed the online questionnaires via the Wenjuanxing (Changsha Ranxing Information Technology Co., Ltd.) platform and received course credit for participation. Participants were also informed of the purpose, procedure, potential risks, confidentiality concerns, and remuneration of the project, as well as their right to withdraw from the project at anytime. Informed consent was obtained from all participants including parents of 45 minor participants. Participation was voluntary; writing summary papers on literature readings and participating in other studies were alternatives for students to obtain credits.

### Measures

#### Mindfulness scale

Chinese version of the Five Facet Mindfulness Questionnaire (FFMQ) was used in the present study, which has shown an acceptable psychometric property for the assessment of mindfulness [37]. This questionnaire evaluates five facets of mindfulness: observing, describing, acting with awareness, non-judging, and non-reacting. Observing refers to noticing or attending to external and internal stimuli. Describing means labelling experience with words. Acting with awareness refers to concentrating on the present situation without distraction. Non-judging refers to accepting, allowing or being nonevaluative to the present. Non-reacting refers to permitting inner thoughts and feeling to come and go without getting stuck in. Examples of items are “I intentionally stay aware of my feelings” and “I find myself doing things without paying attention”. All 39 items are measured by a 5-point Likert scale ranging from 1 = *never* to 5 = *very often*. Participants with higher scores of FFMQ are considered to possess higher mindfulness in daily life. As a rule of thumb, values between 0.70 and 0.80 were regarded as acceptable, and > 0.8 as having good reliability [38]. The Chinese version of FFMQ was initially used in adults and had good reliability (Cronbach α = 0.83) [37]. For this study, the measure demonstrated acceptable reliability (Cronbach α = 0.77).

#### Resilience scale

The Connor-Davidson resilience scale (CD-RISC) is a brief, self-rated measure of resilience [39]. A Chinese version of it has been validated [40], which comprises 25 items, each rated on a 5-point Likert scale (0 = *not at all* to 4 = *extremely*). Those with higher scores reflect better resilience. The Chinese version of CD-RISC was initially used in adolescents and had good reliability (Cronbach α = 0.86) [40]. For this study, the measure demonstrated good reliability (Cronbach α = 0.93).

#### Emotional dysfunction scale

The Chinese version of the Depression Anxiety Stress (DASS-21) Scales [41, 42] was applied to assess the stress, depression and anxiety symptoms within a week. This questionnaire is rated on a 4-point Likert-type scale from 0 to 3. Participants with higher scores may have more serious emotional dysfunction. The Chinese version of DASS-21 was initially used in emerging adults and had good reliability (Cronbach α = 0.92, 0.92, and 0.93 for anxiety, depression, and stress subscales, respectively) [41]. For this study, the measures demonstrated acceptable to good reliability (Cronbach α = 0.74, 0.81, and 0.79 for anxiety, depression, and stress subscales, respectively).

#### Sleep scale

The PSQI is a 19-item self-rated questionnaire designed to measure seven domains to calculate component scores: subjective sleep quality (1 item), sleep latency (2 items), sleep duration (1 item), habitual sleep efficiency (3 items), sleep disturbances (9 items), use of sleeping medication (1 item), and daytime dysfunction (2 items) [43]. Each component has a score that ranges from 0 to 3. The scores of seven components will be summed to yield a PSQI global score ranging from 0 to 21; higher scores indicated poorer sleep quality. Examples of the questionnaire are “During the past month, when have you usually gone to bed at night?” and “During the past month, how often have you taken medicine (prescribed or “over the counter”) to help you sleep?”. A Chinese version of the scale was validated on adults [31]. The Chinese version of PSQI was initially used in adults and had good reliability (Cronbach α = 0.83) [31]. For this study, the measure demonstrated good reliability (Cronbach α = 0.77).

### Statistical analyses

Data entry, management and descriptive statistics were performed using SPSS version 24.0. The descriptive analysis of the main variables was conducted and Pearson’s correlations were also calculated in order to explore the relationship among variables. Common-method variance was determined using Harman’s single-factor test. Next, the plug-in PROCESS and Model 6 were used to provide serial-multiple mediating model results. Firstly, mindfulness was entered as the independent variable, resilience and emotional dysfunction as the first and second mediator variables, and sleep quality as the outcome variable (see Fig. 1). Secondly, this was repeated with five mindfulness facets (observing, describing, acting with awareness, non-judging, non-reacting) as independent variables, resilience as the first mediator variable, three DASS subscales (depression, anxiety, stress) as the second mediator variables, and seven sleep quality components (i.e., subjective sleep quality, sleep latency, sleep duration, habitual sleep efficiency, sleep disturbances, use of sleeping medication, and daytime dysfunction) as outcome variables. The 95% bias-corrected confidence intervals (Cis) of direct and indirect effects were generated by bootstrapping with 5000 resamples [44]. The effects were regarded statistically significant if their Cis did not include 0.

### Common method bias (CMB) test

Common method bias is normally prevalent in studies using self-administered questionnaires [45]. Measuring different constructions with similar scales format (e.g., Likert scales) can lead to spurious effects due to the measurement instruments rather than to the constructs being measured [46]. Harman’s single-factor test is one of the most widely used techniques to address the issue of common method bias [47]. Researchers load all the variables in their study into an exploratory factor analysis, and if a substantial amount of variance is present, either a single factor will emerge from the factor analysis or one general factor will account for the majority of the covariance [47].

### Validation analyses

Some studies found the effects of gender on sleep quality, which may even cause differences in specific components of sleep [14, 48, 49]. Also, the wide disparity between urban and rural residences (including family income, body mass index (BMI) results) may affect sleep habits and health [50, 51]. Therefore, the results were reanalysed by regressing out gender, household registration, and age as co-variates in testing each model.

## Results

### Descriptive statistics

497 participants were eventually included, comprising 180 male and 317 female individuals between middle adolescence and emerging adulthood which ranged from 15 to 23 years old [mean = 18.28; standard deviation (SD) = 0.76, see Table 1]. Descriptive statistics (i.e., means and SDs) and bivariate correlations of the key variables are presented in Table 2. As expected, results showed a positive correlation between mindfulness and sleep quality. Additionally, we also found that mindfulness was positively associated with resilience and negatively associated with emotional dysfunction. Further, resilience was negatively associated with emotional dysfunction and positively associated with sleep quality (*p*_s_<0.05). The correlation between emotional dysfunction and sleep quality was negative.

**Table 1:** Demographic and psychometric variables

	Mean	SD
Age (years)	18.27	0.76
Sex (female/male)	317/180	
Residence (urban/rural)	285/212	
Mindfulness scale		
FFMQ total	114.10	11.72
Observing	24.17	5.16
Describing	25.28	5.10
Acting with awareness	26.95	5.15
Non-judging	22.47	4.53
Non-reacting	21.19	2.92
Resilience scale		
CD-RISC total	60.93	14.36
Emotion regulation scale		
DASS total	10.22	7.95
Stress	4.47	3.26
Anxiety	3.23	2.65
Depression	2.51	2.95
Sleep scale		
PSQI total	5.71	2.90
Subjective sleep quality	0.84	0.68
Sleep latency	1.28	1.34
Sleep duration	0.95	0.65
Habitual sleep efficiency	0.17	0.43
Sleep disturbances	0.90	0.47
Use of sleeping medication	0.01	0.16
Daytime dysfunction	1.57	0.95

Abbreviations: SD, standard deviation; FFMQ, the Five Facet Mindfulness Questionnaire; CD-RISC, the Connor-Davidson resilience scale; DDAS, the Depression Anxiety Stress Scales; PSQI, the Pittsburgh sleep quality index

**Table 2:** Descriptive statistics and correlation analysis results of each variable

	1	2	3	4	5	6	7	8	9	10	11	12	13	14	15	16	17	18	19
1 **FFMQ total**																			
2 Observing	0.479^***^																		
3 Describing	0.772^***^	0.242^***^																	
4 Acting with awareness	0.645^***^	− 0.070	0.423^***^																
5 Non-judging	0.300^***^	− 0.314^***^	0.068	0.294^***^															
6 Non-reacting	0.396^***^	0.283^***^	0.167^***^	0.028	− 0.174^***^														
7 **CD-RISC total**	0.631^***^	0.266^***^	0.572^***^	0.461^***^	0.013	0.347^***^													
8 **DASS-21 total**	− 0.463^***^	0.050	− 0.357^***^	− 0.524^***^	− 0.221^***^	− 0.210^***^	− 0.517^***^												
9 Stress	− 0.424^***^	0.054	− 0.310^***^	− 0.485^***^	− 0.203^***^	− 0.226^***^	− 0.420^***^	0.912^***^											
10 Anxiety	− 0.385^***^	0.092^*^	− 0.305^***^	− 0.448^***^	− 0.219^***^	− 0.181^***^	− 0.423^***^	0.895^***^	0.744^***^										
11 Depression	− 0.431^***^	− 0.007	− 0.344^***^	− 0.472^***^	− 0.173^***^	− 0.154^***^	− 0.548^***^	0.881^***^	0.682^***^	0.690^***^									
12 **PSQI total**	− 0.263^***^	0.025	− 0.173^***^	− 0.376^***^	− 0.102^*^	− 0.078	− 0.333^***^	0.441^***^	0.404^***^	0.416^***^	0.367^***^								
13 Subjective sleep quality	− 0.215^***^	− 0.028	− 0.111^*^	− 0.271^***^	− 0.089^*^	− 0.086	− 0.293^***^	0.317^***^	0.290^***^	0.288^***^	0.274^***^	0.748^***^							
14 Sleep latency	− 0.142^***^	0.009	− 0.111^*^	− 0.196^***^	− 0.026	− 0.038	− 0.168^***^	0.229^***^	0.190^***^	0.239^***^	0.191^***^	0.785^***^	0.559^***^						
15 Sleep duration	− 0.106^*^	− 0.011	− 0.051	− 0.201^***^	− 0.014	− 0.011	− 0.159^***^	0.154^***^	0.150^***^	0.144^***^	0.120^***^	0.457^***^	0.176^***^	0.126^***^					
16 Habitual sleep efficiency	0.022	− 0.004	0.083	− 0.005	− 0.017	− 0.012	− 0.002	− 0.034	− 0.023	− 0.054	− 0.018	0.291^***^	0.146^***^	0.102^*^	0.164^***^				
17 Sleep disturbances	− 0.156^***^	0.082	− 0.127^**^	− 0.242^***^	− 0.091^*^	− 0.065	− 0.223^***^	0.387^***^	0.354^***^	0.368^***^	0.320^***^	0.514^***^	0.344^***^	0.282^***^	0.077	0.049			
18 Use of sleep medication	0.047	− 0.008	0.070	0.016	0.012	0.053	0.056	0.022	0.017	0.042	0.004	0.097^*^	0.000	0.100^*^	0.046	− 0.029	0.044		
19 Daytime dysfunction	− 0.312^***^	0.054	− 0.241^***^	− 0.414^***^	− 0.150^**^	− 0.085	− 0.355^***^	0.508^***^	0.483^***^	0.461^***^	0.419^***^	0.683^***^	0.413^***^	0.286^***^	0.278^***^	0.047	0.346^***^	− 0.046	

Abbreviations: FFMQ, the Five Facet Mindfulness Questionnaire; CD-RISC, the Connor-Davidson resilience scale; DASS-21, the Depression Anxiety Stress Scales; PSQI, the Pittsburgh sleep quality index. N = 497, ***p<0.001, *p<0.05

### Common method bias (CMB) test

The analysis yielded 12 factors with eigenvalue greater than 1, and the variation explained by the first one was 18.27%, which did not exceed the critical standard of 40% [47]. Moreover, these 12 factors explained 61.42% of the total variation, which was acceptable owning to more than the minimum criteria of 50% [52]. Therefore, it suggested that CMB did not pose a serious threat to interpreting our present study.

### Influence of trait mindfulness on sleep quality

**Fig. 2:**
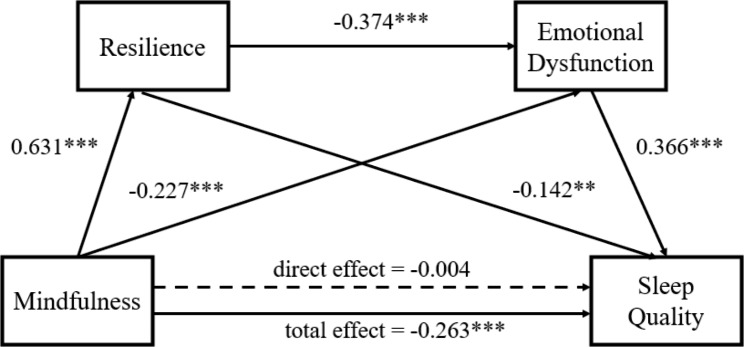
Resilience and emotional dysfunction mediate the association between low mindfulness and poor sleep quality. Paths and path coefficients (standardized) of the mediation models. ^***^p<0.001

Standardized effects between variables are presented in Fig. 2. This study found a positive effect of mindfulness on sleep quality (total effect: *B* = -0.263, SE = 0.043, 95% CI = [-0.348, -0.177], *p* < 0.001). However, when resilience and emotional dysfunction were included in the analysis, this coefficient was not statistically significant (direct effect: *B* = -0.004, SE = 0.053, 95% CI = [-0.108, 0.100], *p* = 0.943). Mindfulness was found associated with resilience (*B* = 0.631, SE = 0.035, 95% CI = [0.563, 0.700], *p* < 0.001) and emotional dysfunction (*B* = -0.227, SE = 0.049, *p* < 0.001).

Effects of each path in the mediation model were listed in Table 3. The present study found a significant indirect effect of mindfulness →resilience →sleep quality (*B* = -0.089, SE = 0.036, 95% CI = [-0.162, -0.023]). In addition, the indirect effect of mindfulness →emotional dysfunction →sleep quality was also significant (*B* = -0.083, SE = 0.019, 95% CI = [-0.123, -0.047]). Finally, the study indicated a remarkable indirect effect of mindfulness →resilience →emotional dysfunction →sleep quality (*B* = -0.086, SE = 0.020, 95% CI = [-0.127, -0.051]). The ratio of total indirect effect and three branches of indirect effects to total effect were 96.1%, 33.4% (mindfulness →resilience →sleep quality), 30.9% (mindfulness →emotional dysfunction →sleep quality) and 31.8% (mindfulness →resilience →emotional dysfunction →sleep quality), respectively.

**Table 3:** Effects of mediation models

Model Paths	*ß*	SE	Bias-corrected CI (95%)		Weight (%)
			Lower	Upper	
Total Effects					
**M**→**SQ**	**-0.263**	**0.043**	**-0.348**	**-0.177**	100
Direct Effects					
M→SQ	-0.004	0.053	-0.108	0.100	3.9
Indirect Effects					
**M**→**R**→**SQ**	**-0.089**	**0.036**	**-0.162**	**-0.023**	33.4
**M**→**ED**→**SQ**	**-0.083**	**0.019**	**-0.123**	**-0.047**	30.9
**M**→**R**→**ED**→**SQ**	**-0.086**	**0.020**	**-0.127**	**-0.051**	31.8

Abbreviations: ***ß***, standardized coefficient; SE, standard error; CI, confidence interval; M, mindfulness; R, resilience; SQ, sleep quality; ED, emotional dysfunction. Significance in bold

**Fig. 3:**
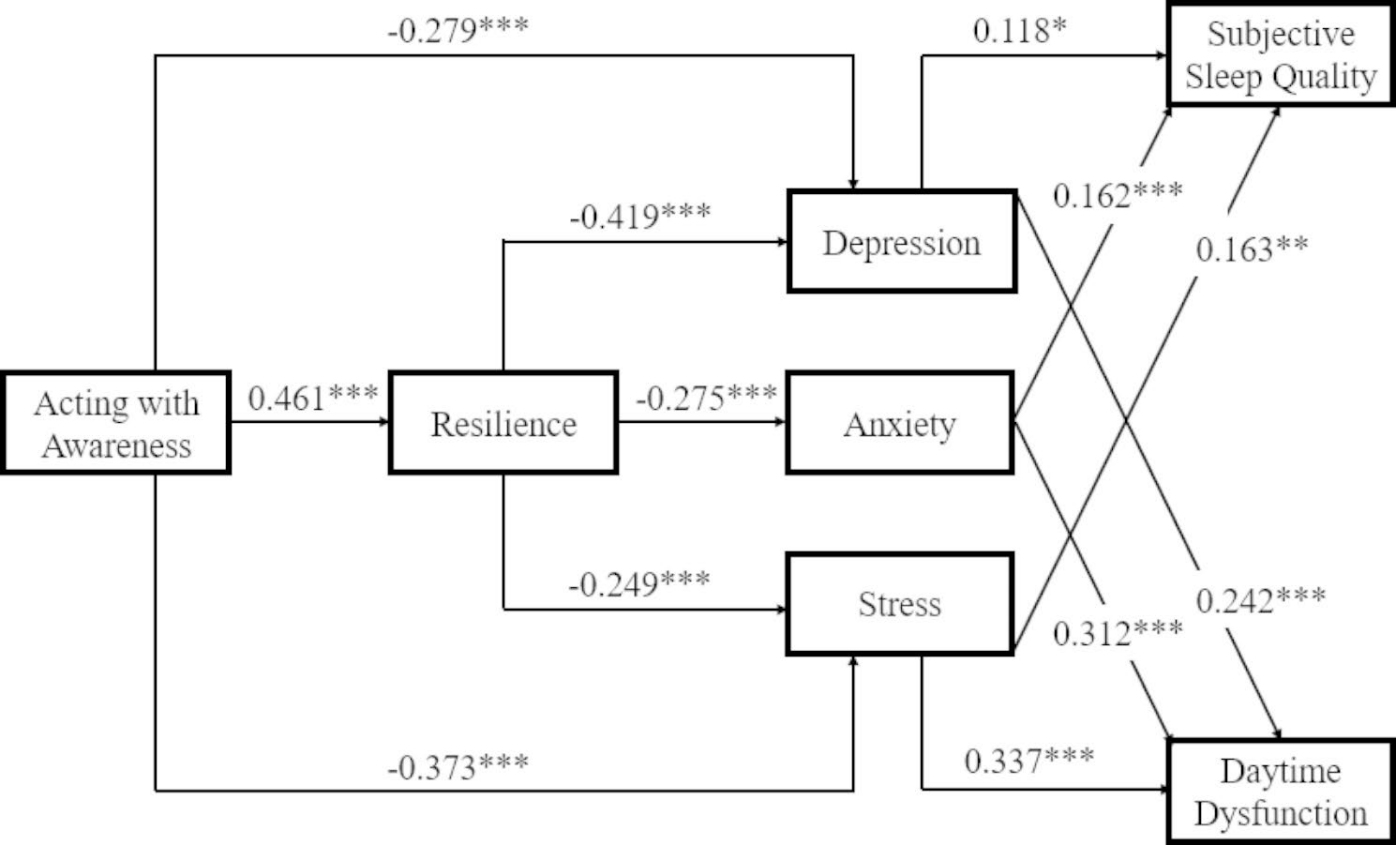
Acting with awareness (mindfulness facet) showed significant indirect effect on subjective sleep quality and daytime dysfunction (sleep quality components) through resilience and emotional dysfunction (depression, anxiety and stress, respectively). Paths and path coefficients (standardized) of sequential mediation models. ^**^p<0.01, ^***^p<0.001

The specific direct and indirect effects of each facet of trait mindfulness on components of sleep quality were displayed in Fig. 3. Among multiple mediation models, acting with awareness (mindfulness facet) showed a significant indirect effect on subjective sleep quality and daytime dysfunction (sleep quality components) through resilience and through emotional dysfunction (depression, anxiety and stress, respectively). Furthermore, acting with awareness also had an indirect effect on these two sleep quality components through resilience and emotional dysfunction (i.e., depression, anxiety and stress) in sequence (see Table S1 in the supplementary). The indirect effects of acting with awareness on the other five sleep quality components (sleep latency, sleep duration, habitual sleep efficiency, sleep disturbances, or use of sleeping medication) were not significant. There is no association between acting with awareness and habitual sleep efficiency, use of medication. Resilience and emotional dysfunction did not mediate the association between acting with awareness and sleep latency, sleep duration. Resilience did not mediate the association between acting with awareness and sleep disturbance.

### Validation analyses

The findings remained even after the exclusion of co-variates (i.e., gender, household registration and age) (see Figure S1 and Figure S2 in the supplementary). Each path only changes numerically, while the significance remains unchanged.

## Discussion

To the best of our knowledge, this is the first study to examine the roles of resilience and emotional dysfunction in the impact of trait mindfulness on sleep quality among individuals between middle adolescence and emerging adulthood. Although the association between trait mindfulness and sleep quality has been noted by previous studies [10, 53, 54], the underlying mechanisms remain largely unclear among individuals between middle adolescence and emerging adulthood who have a high prevalence of poor sleep quality [6]. The present study tested whether trait mindfulness would associate with sleep quality through the modulation of resilience and emotional dysfunction. Importantly, the results demonstrated that the impact of trait mindfulness on sleep quality may be mediated by resilience and emotional dysfunction, after controlling for gender and household registration type. In other words, trait mindfulness was related to high resilience, and in turn, high resilience predicted positive emotion, and positive emotion may attenuate sleep problems. Apart from that, we further explored the associations between mindfulness facets and sleep quality components through the mediation models. Mindfulness facet of acting with awareness and sleep components of subjective sleep quality and daytime dysfunction were found significant in the mediation models. That is, acting with awareness related to high resilience, high resilience was associated with less emotional dysfunction, and thus improving subjective sleep quality as well as daytime dysfunction.

### Mindfulness relates to sleep quality positively

Mindfulness has long been regarded as relating to mental health, possibly for its engagement in attentional control and emotional regulation [55]. Therefore, it is plausible that this psychological process is rather critical for those who suffer from emotional difficulties [14]. Additionally, mindfulness may equip emerging adults with a sense of self-control towards their lives [56]. Encouraging mindfulness in cognitive control and emotion regulation could be applied to emerging adults, which may contribute to mitigating the occurrence of sleep disorders [9, 10, 57]. Indeed, emerging adulthood is a transition period into an adult role [58], during which it might be painful for emerging adults to encounter the ambiguity of being an adult. Therefore, the possibility of the onset and escalation of psychological disorders such as major depression and anxiety disorder increases across puberty maturation [59]. The results offer implications for future research concerning the facets of mindfulness in relation to mental well-being.

The current study extended the literature on the benefits of mindfulness on mental well-being [36, 60, 61]. There were five facets of mindfulness (observing, describing, acting with awareness, non-judging, and non-reacting) among which acting with awareness was confirmed significant in the present study, consistent with former research [34, 35, 57]. Acting with awareness has been described as the central defining core of mindfulness, which may facilitate attention to one’s needs, values, and interests, making one more likely to regulate behaviour that leads to psychological well-being [8]. Emerging adults with their own initiatives to deal with various difficulties may have a sense of self-control and domination towards themselves. Therefore, they may believe in themselves to overcome sleep difficulties, by trying to detach from rather than surrender to various triggers of sleep problems. Higher resilience may enable them to detach from unwanted or intrusive thoughts and switch back to sleep mode in a shorter time [19]. Prior mentioned, there were seven components of PSQI (subjective sleep quality, sleep latency, sleep duration, habitual sleep efficiency, sleep disturbances, use of sleeping medication, and daytime dysfunction) among which the components of subjective sleep quality and daytime dysfunction were found significant in mediation models, respectively. It has also been postulated that resilience and daytime dysfunction could influence each other in the long term which shares a similar mechanism of modulating human’s brain [62, 63].

### The mediating role of resilience

Together with the findings that resilience has direct effects on the occurrence of sleep quality, our findings proposed a potential intervention for emerging adults that is shown to evolve stronger mindfulness. Such an intervention would be prospective in strengthening the ability to overcome sleep problems by raising individual resilience, suggested by Morin’s cognitive-behavioural model [20]. Another research indicated that trait mindfulness may promote resilience by suppressing unrelated information or provoking physiological arousal [61], which may result in fewer sleep disturbances [64, 65].

### The mediating role of emotional dysfunction

The second sequential indirect path was mindfulness negatively associated with emotional dysfunction, while the latter was negatively associated with sleep quality, which mainly consists with prior studies [10, 66]. Mindfulness is deemed as an approach to emotion regulation [67], for individuals with higher levels of mindfulness may have better skills in coping with emotional dysfunction [68]. A study provided longitudinal evidence that mindfulness facet of acting with awareness could significantly predict sleep quality through emotional distress among Chinese cancer patients [69]. Although mindfulness facets of observing and non-reacting indicated a significant link with sleep quality through psychological distress [70], none of such results were detected in the present study. Additionally, some studies have observed that individuals with emotional dysfunction were at a higher risk of sleep dysfunction [71–73]. For instance, social anxiety has been implied associated with specific sleep problems of sleep latency, sleep disturbance, and daytime sleep dysfunction [74]. A potential explanation is that individuals with stress, anxiety or depression are less likely to use adaptive coping strategies, leading to a worsening of sleep quality [75].

### Resilience and emotional dysfunction

The third sequential path was mindfulness positively connected with resilience, then resilience negatively associated with emotional dysfunction, and the latter negatively correlated with sleep quality. Resilience has been confirmed negatively linked to emotional dysfunction according to previous studies [26, 76], possibly explained by that individuals with higher resilience may have stronger problem-solving skills and thus reducing multiple triggers of emotional dysfunction [77]. Apart from that, a longitudinal study indicated that resilience may foster perceived competence and self-confidence [78] which could help encourage individuals to overcome difficulties that may cause emotional distresses.

### Limitations

The current study should be interpreted with caution owing to some existing limitations. Firstly, the cross-sectional design nature of the present study may preclude the exploration of causal correlations among variables. Some relations identified in the current study may be bi-directional or there are additional factors which could mediate or moderate the relationships. For example, neuroticism has been found to moderate the impact of mindfulness on sleep quality in college students [10]. Hence, our findings should be further verified by more longitudinal studies with better control of underlying contributing factors. Secondly, due to potential introduced recall bias, the self-reported approach may limit the validity of the results [47]. Thirdly, the study sample only represented emerging adults from a single university and the results may therefore be unable to generalize to a larger emerging adults population. Future studies involving multiplex samples from varied universities are required. Fourthly, the non-probability sampling methods (self-selection sampling) and volunteer bias (i.e., individuals who have trouble in sleep may be more prone to participate) may undermine the external validity. Therefore, more probability-sampling studies with smaller biases should further inspect the study. Fifthly, we did not include BMI and family income as covariates, which may be cofounders in the study. Scholars indicated that low-income families mainly concentrate in rural areas for natural, geographical, and policy reasons [79, 80]. A study found that the prevalence of obesity is significant between urban and rural residences [81]. Additionally, there is a certain extent of misreporting of BMI [82]. Results may be inaccurate for BMI and family income covariates, though we simplified the BMI and family income to urban-rural disparity for humanistic care and accurate collection.

## Conclusions

Individuals between middle adolescence and emerging adulthood with higher trait mindfulness may have better sleep quality, while resilience and emotional dysfunctions play mediating roles between them. Moreover, individuals between middle adolescence and emerging adulthood with better acting with awareness (facet of mindfulness) may have better subjective sleep quality and less daytime dysfunction through resilience and emotional dysfunction. To improve sleep quality, it is suggested that future research would focus on the use of mindfulness training in sleep improvement, especially through strengthening resilience and reducing emotional dysfunction.

### Electronic supplementary material

Below is the link to the electronic supplementary material.

**Supplementary Material 1**: Table S1, Figures S1 and S2.

## Data Availability

The datasets used and/or analysed during the current study are available from the corresponding author upon reasonable request.
