# Supplementary material for: Low mindfulness is related to poor sleep quality from middle adolescents to emerging adults: a process model involving resilience and emotional dysfunction
Source: BMC Psychiatry. 2023 Aug 28;23:626. doi: 10.1186/s12888-023-05092-1 (PMC10463593; doi:10.1186/s12888-023-05092-1)

**Supplementary**

**Low Mindfulness Is Related to Poor Sleep Quality from Middle Adolescence to Emerging Adults: A Process Model Involving Resilience and Emotional Dysfunction**

**Huaiyuan Zhou**^1+^**, Ziqing Zhu**^1+^**, Xiangang Feng**^1,2*^**, Ruibin Zhang**^1,2*^

1 Cognitive Control and Brain Healthy Laboratory, Department of Psychology, School of Public Health, Southern Medical University, Guangzhou, China,

2 Department of Psychiatry, Zhujiang Hospital, Southern Medical University, Guangzhou, China

^+^Both authors contributed equally to this work.

*Corresponding to:

Xiangang Feng

Department of Psychology, School of Public Health, Southern Medical University.

Email: xgfeng@smu.edu.cn

Ruibin Zhang

Department of Psychology, School of Public Health, Southern Medical University.

Email: [ruibinzhang@foxmail.com](mailto:ruibinzhang@foxmail.com)

**Table S1.** Indirect effects of mediation models between specific components of trait mindfulness and sleep quality.

| **Model Paths** | ***ß*** | **SE** | **Bias-corrected CI (95%)** | |
| --- | --- | --- | --- | --- |
|  |  |  | **Lower** | **Upper** |
| Mediated Effects | | | | |
| **AA→SSQ** | **-0.131** | **0.031** | **-0.194** | **-0.072** |
| **AA→DD** | **-0.164** | **0.027** | **-0.217** | **-0.111** |
| Direct Effects | | | | |
| **AA→SSQ** | **-0.140** | **0.050** | **-0.238** | **-0.042** |
| **AA→DD** | **-0.250** | **0.046** | **-0.341** | **-0.160** |
| Indirect Effects | | | | |
| **AA→R→SSQ** | **-0.075** | **0.026** | **-0.128** | **-0.026** |
| **AA→D→SSQ** | **-0.033** | **0.017** | **-0.066** | **-0.001** |
| **AA→A→SSQ** | **-0.052** | **0.019** | **-0.092** | **-0.018** |
| **AA→S→SSQ** | **-0.061** | **0.021** | **-0.103** | **-0.021** |
| **AA→R→D→SSQ** | **-0.023** | **0.012** | **-0.047** | **-0.001** |
| **AA→R→A→SSQ** | **-0.021** | **0.009** | **-0.039** | **-0.006** |
| **AA→R→S→SSQ** | **-0.019** | **0.007** | **-0.035** | **-0.006** |
| **AA→R→DD** | **-0.050** | **0.024** | **-0.098** | **-0.003** |
| **AA→D→DD** | **-0.067** | **0.016** | **-0.100** | **-0.037** |
| **AA→A→DD** | **-0.100** | **0.019** | **-0.141** | **-0.066** |
| **AA→S→DD** | **-0.125** | **0.022** | **-0.169** | **-0.084** |
| **AA→R→D→DD** | **-0.047** | **0.012** | **-0.071** | **-0.025** |
| **AA→R→A→DD** | **-0.040** | **0.011** | **-0.062** | **-0.021** |
| **AA→R→S→DD** | **-0.039** | **0.010** | **-0.059** | **-0.022** |

Abbreviations: ***ß***, standardized coefficient; SE, standard error; CI, confidence interval; AA, acting with awareness; R, resilience; D, depression; A, anxiety; S, stress; SSQ, subjective sleep quality; DD, daytime dysfunction. Significance in bold.

**Figure S1.** Resilience and emotional dysfunction mediate the association between low mindfulness and poor sleep quality after the exclusion of co-variates. Paths and path coefficients (standardized) of the mediation models. ^***^*p*＜0.001.


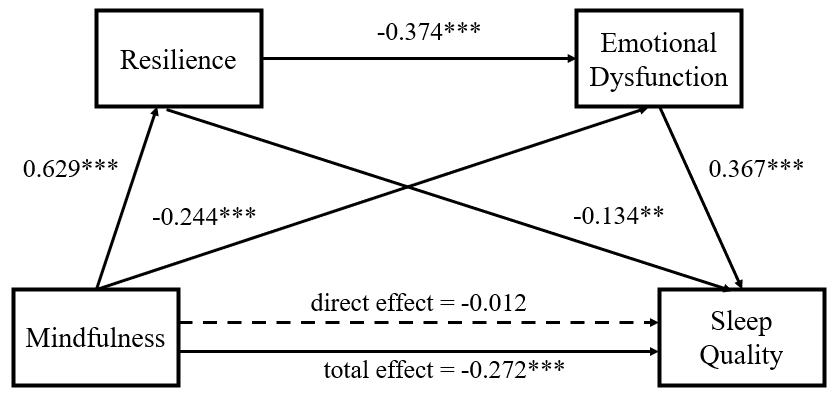


**Figure S2.** Acting with awareness (mindfulness facet) showed significant indirect effect on subjective sleep quality and daytime dysfunction (sleep quality components) through resilience and emotional dysfunction (depression, anxiety and stress, respectively) after the exclusion of co-variates. Paths and path coefficients (standardized) of sequential mediation models. ^**^*p*＜0.01, ^***^*p*＜0.001.


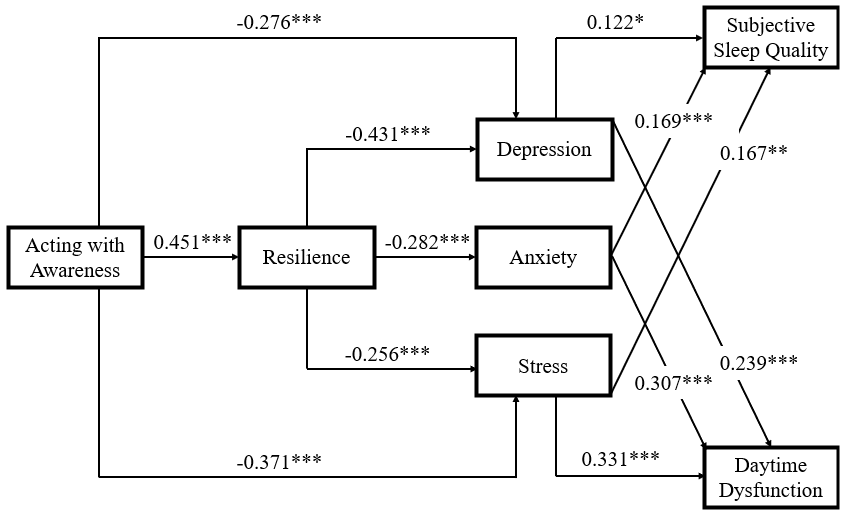

Supplement: Supplementary file 1 — Supplementary Material 1: Table S1, Figures S1 and S2. [file 12888_2023_5092_MOESM1_ESM.docx]
